# Mr. O'Beirne

**Published:** 1825-01-01

**Authors:** 


					SURGEON O'BEIRNE.
'?VSs ?"nesday, the 8th of December, died, after a lingering and painful
?^d.' rp ." O'Beikne, an army surgeon, at his lodgings in the Edgeware
^rve(J ? was a m?st promising and enterprising young gentleman. He
P0Vot.nlt* disastrous campaign against the Ashantees, and was close to the
O ^en he fell into the hands of the barbarians. In that campaign,
]j ^ ^ the jungle fever, which afterwards assumed the intermittent type,
lrassed him till his death. He was attended by Dr. Forbes of Argylo
?eo Unremitting assiduity, and latterly by Dr. James Johnson and
Wacan. These gentlemen examined the body after death. The
Was extreme- The right side of the chest, before and after death,
Rppose ^1? sound, except under the clavicle, which led Doctor Johnson to
f fe w ? *n addition to enlarged and diseased liver, (which was evident,)
^ri(} hepatization of the right lung. This was not the case; but it was
railed ^at S^e was a^most annihilated, but still the small portion that
fr w'as sound. Nearly four pints of a bad purulent matter were eVa-
alSo?m ^le abscess, and the liver was immensely enlarged. The spleen
>0 ' greatly enlarged and, with the liver, encroached on the left side of
o s0Und *' '^e diagnostic error into which Dr. Johnson was led, from the want
Vg m tlle right side of the chest, was very pardonable, since, although
( Ss. n0t ^pdtized, its place was usurped by diseased livor and an,
1C'1 Came to nearly the same thing, as far as the diagnosis by per-
V(), TT Concerned.
L- U. No. 3. 2 L

				

